# Incidence of Cardiac Manifestations in Children with Dengue Fever: A Cross-sectional Study

**DOI:** 10.5041/RMMJ.10436

**Published:** 2021-04-29

**Authors:** Janakiraman Abhinayaa, Saji James, Rathinasamy Jebaraj, Ponnurangam Nagarajan Vinoth

**Affiliations:** 1 Department of Pediatrics, Sri Ramachandra Medical College and Research Institute, Chennai, Tamil Nadu, India; 2 Department of Cardiology, Sri Ramachandra Medical Centre, Chennai, Tamil Nadu, India

**Keywords:** Cardiac, CPK-MB, creatine phosphokinase, myocardial band, dengue, echocardiogram, electrocardiogram

## Abstract

**Objective:**

The aim of our study was to explore the incidence of cardiac involvement in children with dengue infection admitted in a tertiary care hospital and to evaluate the features of cardiac involvement with the severity of dengue fever.

**Methods:**

This was a cross-sectional study conducted from September 2014 to August 2016. A total of 130 patients with confirmed dengue NS1 antigen or IgM antibody positivity between the ages of 1 month and 18 years were evaluated. On the third day of admission, blood samples for cardiac markers were collected, and electrocardiograms (ECG) and echocardiograms were performed for each patient.

**Results:**

Of the 130 dengue patients in the study, 60 (46.2%) were males and 70 (53.8%) were females (male to female ratio, 1:1.16). Cardiac involvement was present in 60 (46.2%) children and was more prominent in children with severe dengue (72.7%), followed by dengue with warning symptoms (53.8%) and dengue fever (28.6%). There was no significant correlation between cardiac involvement and primary/secondary dengue. Both ECG and echocardiography changes were significantly correlated with dengue severity, as opposed to cardiac markers.

**Conclusions:**

Cardiac involvement was present in children with dengue. Evaluation with ECG, echocardiography, and cardiac markers such as creatine phosphokinase–myocardial band (CPK-MB) are required for the management of cardiac complications in children with dengue. Our study showed an association between cardiac involvement and the severity of dengue. Further studies should be framed, and follow-up of dengue patients with cardiac involvement is necessary for therapeutic management.

## INTRODUCTION

Dengue fever is a major health concern in India. Dengue is a mosquito-borne viral infection that causes significant morbidity in endemic regions, with 96 million cases clinically reported annually.^1^ The causative agent is a dengue virus (DENV, 1–4 serotypes), which is a highly prevalent arbovirus found in tropical and subtropical regions.^2^ The first case of dengue in India was reported in 1956 in Vellore, and the first case of dengue hemorrhagic fever was observed in Calcutta in 1963.^3^ The annual incidence of dengue in India has been estimated to be around 7.5–32.5 million,^4^ and it is one of the leading causes for hospitalization and death in India.^5^ According to the World Health Organization (WHO), there has been an increase in reporting of dengue cases for the past five decades.^3^ Dengue infection is prevalent in a majority of the states in India.^6^ Along with an increase in dengue incidence, atypical manifestations of dengue are on the rise, and it is likely to be underreported.^7^

The clinical manifestations in dengue range from asymptomatic infection to severe viral hemorrhagic fever as a prelude to plasma leakage and bleeding.^3^,^8^ However, during defervescence, plasma leakage is reversed and the extravasated fluid is reabsorbed, which is a prelude to fluid overload and reflected by the development of massive pleural effusion or pulmonary edema. Thus, the resulting respiratory manifestations have been the major cause of mortality in adults and children with severe dengue.^9^,^10^ Nevertheless, the cardinal mechanism of shock is due to hypovolemia, and the impaired cardiac function might also contribute to cardiac abnormalities. Several clinical studies have shown the existence of cardiac co-morbidity in dengue.^11^–^13^ Clinically, cardiac involvement can differ broadly, from subclinical to severe myocarditis which can be fatal. Myocardial involvement may be attributed to direct viral invasion or cytokine-induced immune damage, or both. Nevertheless, research on cardiac manifestations of dengue is limited in the pediatric population.

Reports from different studies have shown a 16.7%–71% incidence of cardiac involvement with features like cardiac failure, elevated cardiac enzymes (e.g. troponin T, creatine phosphokinase–myocardial band [CPK-MB]), abnormal electrocardiogram (sinus tachycardia, sinus bradycardia, T wave inversions, heart block), and echocardiogram changes (reduced ejection fraction).^14^,^15^ The variation in symptoms can be attributed to the different criteria used for defining cardiac manifestations.

“Expanded dengue syndrome” is a newly structured class by WHO, comprising unusual manifestations with organ involvement.^16^

The aim of this study was to evaluate if cardiac involvement was present in children with dengue fever. The primary objective of the study was to determine the incidence of cardiac involvement in pediatric patients with dengue, dengue with warning signs, and severe dengue. The secondary objective of the study was to identify a correlation between the clinical cardiac findings and the investigations done in these children with dengue fever.

## MATERIALS AND METHODS

This cross-sectional study was conducted in our tertiary care center in Chennai, Tamil Nadu, from September 2014 until August 2016. Pediatric patients between the ages of 1 month and 18 years with wide clinical presentation of dengue infection, together with subsequent positive dengue NS1 antigen and/or IgM MAC ELISA tests, were included in the study. Children with existing congenital or acquired heart diseases and other coexisting disease conditions were excluded from the study. With an expected 10% of cardiac involvement based on previous studies and with a precision of 5%, confidence interval of 95%, a sample size of 130 was calculated.

### Data Collection/Research Instrument

Patients with clinically suspected dengue fever were administered dengue NS1 (fever lasting <1 week) or dengue IgM serology (fever lasting >1 week) tests. Patients identified with dengue NS1 or as dengue IgM-positive were grouped according to WHO criteria^3^ as having dengue fever, dengue with warning signs, and severe dengue (Table 1).^3^ Patients who were dengue NS1/IgM-positive, but dengue IgG-negative, were considered as primary dengue patients, while those who tested dengue NS1/IgM-positive and dengue IgG-positive were considered secondary dengue patients.

**Table 1:** WHO’s 2009 Dengue Case Classification.^3^.

Severity	Symptoms
Dengue Fever	Fever and two of the following: Nausea, vomiting Rash Aches and pains Leukopenia Positive tourniquet test
Dengue Fever with Warning Signs	Dengue fever as defined above with any of the following: Abdominal pain or tenderness Persistent vomiting Clinical fluid accumulation (ascites, pleural effusion) Mucosal bleeding Lethargy, restlessness Liver enlargement >2 cm Laboratory: increased hematocrit concurrent with rapid decrease in platelet count
Severe Dengue	Dengue fever with at least one of the following criteria: Severe plasma leakage leading to shock Fluid accumulation with respiratory distress Severe bleeding as evaluated by clinician Severe organ involvement ○ Liver Enzymes: AST or ALT ≥1000 U/L ○ Central nervous system: impaired consciousness ○ Failure of heart and other organs

ALT, alanine aminotransferase; AST, aspartate aminotransferase.

All patients were clinically examined and assessed for the following cardiac manifestations: bradyarrhythmia, tachyarrhythmia, pericardial rub, presence of gallop, regurgitation (murmur grade >2/6), and capillary filling time (CFT). On the third day of admission, the following were performed: electro-cardiogram (ECG), two-dimensional echocardiography (ECHO), and serum glutamic oxaloacetic transaminase (SGOT) and creatine phosphokinase–myocardial band (CPK-MB) tests.

Based on reports from previous studies, cardiac involvement was considered present if dengue-positive (NS1/IgM) patients exhibited any two or more of the following factors: elevated CPK-MB, abnormal ECG findings, and/or abnormal echocardiography findings.^13^–^15^ Those patients with features suggestive of cardiac involvement were placed on follow-up with the pediatric cardiologist.

The CPK-MB estimation was performed for all patients who tested positive for dengue. This was done by immuno-inhibition method.^17^ Similarly, SGOT estimation was performed by the indirect enzymatic method.^18^

Electrocardiography was performed on all dengue-positive patients. Smaller size ECG leads were used in patients less than 2 years of age. Cardiac involvement was determined from the ECGs based on the presence of any of the following features: ST-segment elevation in ≥2 contiguous leads (>1 mm in limb leads, and >2 mm in chest leads), T wave inversions in V5 and V6, widespread ST-segment depressions (sustained horizontal ST-segment depression for 0.08 seconds or longer), and pericardial involvement such as a low-voltage QRS complex (QRS <5 mm in limb leads and <10 mm in the chest leads), ST-elevation with concavity upwards (>1 mm in limb leads, and >2 mm in chest leads), or T wave inversion in V5 and V6.^19^

Echocardiography was performed on all dengue-positive patients using a Vivid 7 machine (Wipro GE Healthcare Pvt Ltd, Bangalore, Karnataka, India; date of manufacture 2007). Cardiac manifestations included left ventricular dysfunction (left ventricular ejection fraction <55%), left ventricular cavity enlargement (according to body size/weight), right ventricular dysfunction (pulmonary artery systolic pressure estimated using tricuspid regurgitation, right ventricular systolic function visual inspection), segmental wall motion abnormalities (hypokinetic, akinetic, or dyskinetic regions), and pericardial findings such as pericardial effusion (mild, <100 mL; moderate, 100–500 mL).^20^

### Ethical Approval

This study was approved by the Institutional Ethics Research Committee of Sri Ramachandra Institute of Higher Education and Research (Deemed to be University) Ref: CSP-MED/14/SEP/18/153. The purpose of the study was explained, and written informed consent was obtained from caretakers or parents of the children; in addition, assent was taken in children more than 12 years of age.

### Statistical Analysis

The data were entered in Microsoft Excel and then transferred to SPSS software (version 17.0) for further statistical analysis. The results were represented as categorical data, and chi-square test was used. We reported statistically significant *P* values (*P*≤0.05) and their 95% confidence intervals.

## RESULTS

Among the total 130 dengue patients, most of the patients were 5–9 years of age (*n*=26, 43.3%) (Table 2). There were 60 (46.2%) males and 70 (53.8%) females present, with a higher proportion of females in this study.

**Table 2:** Cardiac Involvement by Age Group in the Study Group (*n*=130).

Age Group (Number)	With Cardiac Involvement	Percent	*P* Value
Overall (*n*=130)	60	46.2%	0.077
<1 year (*n*=20)	6	30%	
1–4 years (*n*=26)	12	46.2%	
5–9 years (*n*=41)	26	63.4%	
10–14 years (*n*=29)	11	37.9%	
15–18 years (*n*=14)	5	35.7%	

**Figure 1:**
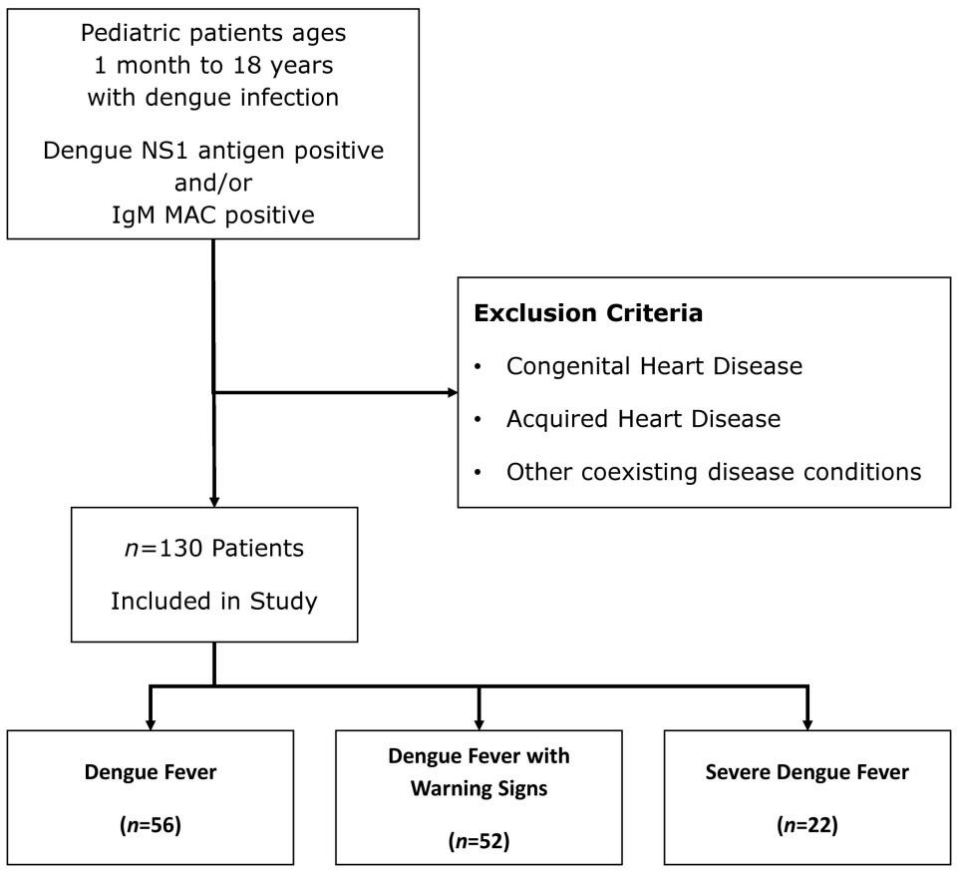
Flowchart for Identification of Children with Dengue Infection in This Study.

The final study group comprised 56 (43%) patients with dengue fever, 52 (40%) with dengue with warning signs, and 22 (17%) with severe dengue (Figure 1). In the latter two groups, the majority of patients were 5–9 years of age. Referring to Table 3, the highest percentage of cardiac manifestations was noted in the severe dengue group (*n*=16, 72.7%), followed by the dengue with warning signs group (*n*=28, 53.8%).

**Table 3:** Cardiac Involvement in Dengue Patients.

Category (*n*)	With Cardiac Involvement *n* (%)	Without Cardiac Involvement *n* (%)
Dengue fever (56)	16 (28.6)	40 (71.4)
Dengue with warning signs (52)	28 (53.8)	24 (46.2)
Severe dengue (22)	16 (72.7)	6 (27.3)
Total (130)	60 (46.2)	70 (53.8)

There was a significant association (*P*=0.005) between the length of hospital stay and dengue patients with cardiac involvement as compared to dengue patients without cardiac involvement (Table 4). Length of hospitalization was higher in dengue patients with cardiac involvement (Table 4).

**Table 4:** Duration of Hospital Stay and Cardiac Involvement.

Hospital Stay Duration	Patients with Cardiac Involvement, *n* (%)	Patients without Cardiac Involvement, *n* (%)	*P* Value*
0–4 days	2 (4.8)	39 (95.2)	0.005
4–8 days	45 (62.5)	27 (37.5)	
8–13 days	13 (76.4)	4 (23.6)	
Total *n* (%)	60 (46.2)	70 (53.8)	

* *P* value determined using chi-square test.

No significant difference was noted between the primary and secondary dengue patients with cardiac involvement (*P*=0.337) (Table 5).

**Table 5:** Incidence of Cardiac Involvement in Primary and Secondary Dengue.

Dengue Type/Severity	Primary Dengue			Secondary Dengue		
	With Cardiac Involvement *n*	Without Cardiac Involvement *n*	Total *n*	With Cardiac Involvement *n*	Without Cardiac Involvement *n*	Total *n*
Dengue fever	9	31	40	7	9	16
Dengue with warning signs	15	17	32	13	7	20
Severe dengue	13	4	17	3	2	5
Total *n* (%)	37 (41.5)	52 (58.4)	89	23 (56.1)	18 (43.9)	41

Among the 130 dengue patients in this study, 62 (47.7%) had ECG changes, the majority of which belonged to the dengue with warning signs group. A significant correlation was noted between the ECG findings and the severity of dengue (*P*=0.022) (Table 6). These findings included sinus bradycardia *n*=11 (17%), of which 6 patients (11.5%) had dengue fever with warning signs. Forty-four (70%) children had T wave inversions (V5, V6), 22 (42.3%) of whom belonged to the dengue with warning signs group.

**Table 6:** Correlation between Cardiac Findings and Dengue Severity.

Cardiac Findings	Dengue Fever (*n*=56)	Dengue with Warning Signs (*n*=52)	Severe Dengue (*n*=22)	Total (*n*=130)	*P* Value*
ECG					0.022
ECG changes	19 (33.9)	31 (59.6)	12 (54.5)	62 (47.7)	
Normal ECG	37 (66.1)	21 (40.4)	10 (45.5)	68 (52.3)	

Echocardiogram					<0.0001
LVEF <55%	1 (1.8)	2 (3.8)	1 (4.5)	4 (3.1)	
Pericardial effusion	6 (10.7)	7 (13.5)	10 (45.5)	23 (17.7)	

CPK-MB					0.138
Elevated	36 (64.3)	42 (80.8)	17 (77.3)	95 (73)	
Normal	20 (35.7)	10 (19.2)	5 (22.7)	35 (27)	

* Test applied: chi-square test.

CPK-MB, creatine phosphokinase–myocardial band; ECG, electrocardiogram; LVEF, left ventricular ejection fraction.

One patient with severe dengue and one with dengue with warning signs had pathological Q waves and a narrow QRS complex, respectively. ST-elevation/depression was observed in 5 (8%) participants, of whom 3 (5.4%) had dengue fever.

Of the 130 dengue patients, 27 had abnormal echocardiography findings, including pericardial effusion (*n*=23, 85%) followed by left ventricular ejection fraction <55% (*n*=4, 15%). On analysis, echocardiography findings for cardiac involvement were significantly associated with the severity of dengue (*P*<0.0001).

Levels of CPK-MB were elevated in 95 (73%) patients. However, there was no significant correlation (*P*=0.138) between the CPK-MB levels and severity of dengue in our study, as shown in Table 6. Among the 95 patients with high CPK-MB levels, 47 (49.4%) had abnormal ECG findings and 21 (22.1%) had echocardiography changes (not shown in tables).

Elevated SGOT levels were found in 54.7% of dengue patients with cardiac involvement and were statistically significant (*P*=0.025). Also, CFT prolongation was present in 5 (100%) participants with cardiac involvement, while none were reported in participants without cardiac involvement in our study. Thus, CFT prolongation was significantly correlated with cardiac involvement (*P*=0.014).

## DISCUSSION

Cardiac manifestations in patients with dengue fever include cardiac failure, ECG changes, 2D ECHO changes, and elevated cardiac enzymes.^15^,^21^ There is a paucity of data on cardiac involvement in dengue infection; however, there is an increasing trend of cardiac involvement in dengue patients being reported. The clinical severity of dengue varies with age. Younger children with dengue hemorrhagic fever elicit more severe clinical outcomes and a higher fatality rate compared to adults.^8^,^22^,^23^ In this study, a higher proportion of children affected with dengue were in the 5–9 years age group, similar to the observations of Salgado and others.^24^,^25^

There have been reports that the increased resting diastolic calcium ion levels present in the myocardium precipitated by dengue can be attributed to the arrhythmia and diminished left ventricular function noted in these patients.^24^ In our study, the overall cardiac involvement among dengue patients in the pediatric population was 46.2%, predominantly in the severe dengue group, similar to the study conducted by Siddappa et al.^26^ The most common features suggestive of cardiac involvement noted in our study were T wave depression and pericardial effusion. Severity of dengue was also directly proportional to the length of hospitalization. Similar findings have been reported by Mishra et al.^27^

Severe cardiac manifestations have been reported in secondary dengue infections.^28^ In our study, cardiac involvement was higher in secondary compared with primary dengue cases, but no statistical significance was noted.

We also found elevated CPK-MB levels in the majority of dengue patients. The level of CPK-MB is an independent marker of myocardial damage in children with dengue infection.^13^ A similar study by Salgado et al. reported that myocarditis was significantly correlated with dengue severity.^24^ However, our study found no statistically significant correlation between CPK-MB and dengue severity.

Abnormalities in ECG tend to occur during any phase of dengue illness, and reports suggest that 30%–44% of dengue patients require hospitalization.^29^ In this present study, the abnormal ECG findings in dengue patients with cardiac involvement were T wave inversions, sinus bradycardia, ST-elevation/depression, pathological Q waves, and changes to the QRS complex.^15^,^21^ A higher proportion of T wave depression was observed in our study. However, Siddappa et al. reported a higher incidence of sinus tachycardia, while T wave inversions were seen in a small number of patients, which was comparatively lower when compared to our study.^26^ These abnormalities are benign, and such ECG abnormalities may be the only sign of cardiac involvement with normal biomarker levels and echocardiogram. Arrhythmias have been seen in severely ill dengue patients, and these patients are more likely to develop hypotension than those having a normal ECG. Electrolyte and calcium changes, altered autonomic tone, or subclinical myocarditis are the possible mechanisms for ECG abnormalities in dengue fever.^30^ In our study, children with abnormal ECG findings were monitored until discharge. They were managed conservatively and did not require any active intervention.

Abnormal echocardiography findings were more prevalent among severe dengue patients in our study. These findings are consistent with earlier studies showing that cardiac functional abnormalities in echocardiography are significantly associated with disease severity.^28^,^31^ In a study performed by Satarasinghe et al., echocardiograph abnormalities were present in 24% of patients, with none having clinical features of overt myocarditis.^32^ Increased cytokine production causes increased vascular permeability and abnormal leakage of plasma, leading to pericardial effusion. The possible mechanism for reduced left ventricular ejection fraction was immune-mediated and direct viral invasion of cardiac muscle cells in myocarditis.^33^ Another finding in our study was left ventricular ejection fraction <55%. But there was no significant correlation between elevated CPK-MB levels and echocardiography findings. In contrast, reports from Salgado et al. revealed a significant association of CPK-MB elevation with myocarditis.^24^ In our study, pericardial effusion resolved as the patients recovered, and pericardiocentesis was not required. One patient had an extended intensive care unit stay and required inotropic support. The rest of the children with pericardial effusion were monitored and managed conservatively (including intravascular volume replacement with judicious care to avoid fluid overload and administration of inotropes and diuretics when necessary).

Elevation of the hepatic marker enzyme, SGOT, was observed and significantly correlated to dengue children with cardiac involvement, similar to several previously published studies.^27^,^34^ Kularatne et al. have reported three cases of dengue where there was multi-organ involvement, with one case presenting in shock.^35^

A significant proportion of dengue patients have cardiac involvement. Evaluation for cardiac involvement in pediatric patients with dengue fever should be performed via ECG, ECHO, CPK-MB, and other similar tests, and would help in the early identification of myocarditis and interventions to prevent further complications.

## LIMITATIONS

There are a few limitations related to this study. Our study was done in a tertiary care center, hence the prevalence of cardiac manifestations we obtained in children with dengue fever cannot be generalized to the entire population. Furthermore, this study included only hospitalized children and did not include children with dengue treated via the outpatient services. In addition, there was insufficient follow-up of children with cardiac involvement: all 60 children with cardiac involvement recovered well and were discharged. Of the patients with cardiac involvement, only 10 were followed, as others were not willing to undergo a repeat evaluation. All 10 patients had normal cardiac evaluation and echocardiography findings 1–3 months after their hospitalization. This, however, was beyond the scope of this study.

## CONCLUSION

We conclude that a significant proportion of dengue patients present with cardiac manifestations; however, most findings are subclinical and do not require further clinical interventions. The predominance of cardiac involvement in dengue patients among children suggests that cardiac screening in all patients with dengue infection, with ECG, echocardiogram, and CPK-MB tests, contributes to effective treatment of myocarditis. This study reports an association of cardiac involvement with the severity of dengue infection in children. Additional large multicenter studies are recommended to further study the acute and long-term impact of dengue infection on the cardiovascular system.

## Abbreviations

CFR: capillary filling time
CPK-MB: creatine phosphokinase–myocardial band
ECG: electrocardiogram
ECHO: echocardiography
SGOT: serum glutamic oxaloacetic transaminase
WHO: World Health Organization.
